# Systematic Review of Studies Comparing Microleakage After Restoration With Cention and Conventional Glass Ionomer Cement in Human Extracted Teeth

**DOI:** 10.7759/cureus.91848

**Published:** 2025-09-08

**Authors:** Rashmi Misra, Mansi Vandekar, Gayatri Pendse, Omkar Bhosale, Pauravi Hegde, Aashaka Vaishnav

**Affiliations:** 1 Conservative Dentistry and Endodontics, D.Y. Patil University School of Dentistry, Navi Mumbai, IND; 2 Dentistry, D.Y. Patil University School of Dentistry, Navi Mumbai, IND

**Keywords:** alkasites, cention, composite resin, dental restoration, restorative dentistry

## Abstract

Cention is an amalgamation of composite resin and glass ionomer cement (GIC) with alkaline fillers, having superior mechanical and aesthetic properties. While the material has proven excellent in providing functional and aesthetic outcomes, its long-term performance in terms of microleakage needs to be studied. The present systematic review was conducted to analyze the currently available evidence concerning the microleakage of Cention and how it fares against GIC in the clinical scenario. A systematic search was performed across various electronic databases, including PubMed, Scopus, Web of Science, and Google Scholar, using the keywords “Cention”, “glass ionomer cement”, and “microleakage”. In-vitro studies assessing microleakage in human extracted teeth after placement of Cention and comparing it to that of GIC were included in the review. Data extraction was performed. The extracted data included study characteristics (authors, publication year, study design), samples used for the study, types of interventions (Cention and conventional GIC), preparatory details (sectioning, thermocycling), and microleakage assessment methods, outcomes, and conclusive findings. The QUIN tool was used for assessing the risk of bias. Based on the conclusive findings presented by the authors of the studies included in the current systematic review, it was apparent that Cention exhibited lower microleakage when compared to GIC. Meta-analysis revealed that the reduction in microleakage on average was 3.12 times more in the Cention group compared to the conventional GIC, and the difference was not statistically significant (p>0.05). The majority of the included studies in the present systematic review found that Cention tended to have lower microleakage compared to conventional GIC, supporting its effectiveness in reducing leakage at the tooth-restoration interface. Our meta-analysis indicates a trend toward reduced microleakage in the Cention group. However, the lack of statistical significance and the presence of substantial heterogeneity and publication bias highlight the need for cautious interpretation.

## Introduction and background

The essence of restorative dentistry lies in repairing the structures of teeth that have been compromised due to dental caries, fractures, or other pathological reasons. The significance of this field lies in its commitment to restoring the functionality and aesthetics of affected teeth, thereby enhancing a patient's overall oral health and quality of life [1]. Thus, an integral factor contributing to the success of dental restorations is the meticulous selection of appropriate dental materials. The choice of materials significantly influences the longevity and effectiveness of the treatment. Among the myriad options available, glass ionomer cements (GICs) have garnered attention for their unique attributes, such as the controlled release of fluoride, biocompatibility, and the inherent ability to form strong chemical bonds with dental tissues [2]. Over the years, GICs have proven themselves as reliable choices in dental practice, appreciated for their capacity to establish a durable seal at the interface between the tooth and the restoration [3].

Even so, the critical issue of microleakage is associated with restorative materials, and conventional GICs are no exception. Microleakage is characterized by the seepage of fluids, bacteria, and ions into the tooth-restoration interface, which leads to the development of secondary caries [4,5]. To effectively tackle the issue of microleakage, continuous research has been taking place over the years in the field of dental materials.

One such modification of conventional GIC is Cention, an alkali-resin-modified GIC. The combination of composite resin and GIC imparts superior mechanical and aesthetic properties to the material [6]. The alkaline filler increases the release of hydroxide ions to regulate the pH value when the environment becomes acidic. While the material has proven excellent in providing functional and aesthetic outcomes, its long-term performance in terms of microleakage needs to be studied [7]. Whether the amalgamation of hydrophobic resins with GIC and the presence of alkaline fillers result in an improvement or worsening of the microleakage is a question to be answered.

However, despite multiple in-vitro evaluations of restorative materials, there remains no clear consensus as to whether modifications such as Cention actually reduce microleakage compared to conventional GICs. This lack of clarity highlights a critical research gap, as minimizing microleakage is directly linked to preventing secondary caries, reducing restoration failure, and improving long-term treatment outcomes in daily clinical practice. The present systematic review was thus conducted to analyze the currently available evidence concerning the microleakage of Cention and how it fares against GIC in the clinical scenario. The objective of the review is to compare the clinical performance of Cention and conventional GIC. The findings of the review would aid dental professionals in the decision-making process when factors such as microleakage are of crucial importance, ultimately leading to improved dental care for patients.

## Review

Methodology

Study Protocol Development and Registration

Before conducting the systematic review, a detailed protocol was developed and registered in the National Institute for Health Research PROSPERO International Prospective Register of Systematic Reviews (registration number: CRD42023463652). The review was planned, performed, and reported according to the Preferred Reporting Items for Systematic Review and Meta-Analyses guidelines (PRISMA) 2020 [8]. The central research question addressed in this systematic review was: “Among in-vitro studies conducted on human extracted teeth, how does the microleakage of restorations placed with Cention compare to those placed with conventional glass ionomer cement?”

Search Strategy

A comprehensive and systematic search strategy was devised to identify all relevant in vitro studies analyzing microleakage in dental restorations using Cention in comparison to conventional GIC. The literature search was conducted across four major electronic bibliographic databases: PubMed (via NCBI), Scopus (via Elsevier), Web of Science Core Collection (via Clarivate Analytics), and Google Scholar. Each database was searched from January 2000 to June 2025. The search was also extended to include gray literature sources such as institutional repositories, electronic theses and dissertations, and relevant conference abstracts, to minimize publication bias. In addition, manual searching of reference lists of all included articles and relevant systematic reviews was performed to identify any potentially eligible studies not captured in the database searches. Gray literature screening included Google Scholar, OpenGrey, and ProQuest Dissertations & Theses Global.

The search strategy incorporated both Medical Subject Headings (MeSH) and free-text terms related to the intervention and outcome. The primary keywords used included combinations and Boolean operators such as: (“Cention” OR “Cention N” OR “alkasite”) AND (“glass ionomer cement” OR “GIC”) AND (“microleakage” OR “marginal leakage” OR “dye penetration”). Filters were not applied to the study design during the initial search phase to ensure inclusivity. The search syntax was adapted to match the indexing format and functionalities of each database. For example, in PubMed, the search was refined using field tags (Title/Abstract) and MeSH terms, while in Scopus and Web of Science, the keywords were searched in titles, abstracts, and keywords fields. In Google Scholar, the top 150 results sorted by relevance were screened manually. A sample search string for PubMed is provided in the Appendix.

Following the database search, all records were exported into a systematic review manager (Elicit Pro), and duplicates were removed. Two independent reviewers then conducted a blinded and structured screening of titles and abstracts to assess the relevance of the studies based on predefined inclusion criteria. Full-text articles of potentially eligible studies were retrieved and evaluated for inclusion.

Selection Criteria

The eligibility of studies was determined using the PICOS (Population, Intervention, Comparison, Outcome, Study Design) framework to ensure clarity and uniformity in the selection process (Table 1). The review focused exclusively on in-vitro studies that evaluated microleakage in human extracted teeth following restoration with Cention and conventional GIC. Studies had to report quantitative microleakage data using validated methods such as dye penetration or stereomicroscopic assessment. Studies were included if they fulfilled the following criteria: use of extracted human teeth, comparison between Cention and conventional GIC as restorative materials, clear methodology for cavity preparation and microleakage evaluation, and in-vitro study design. Exclusion criteria encompassed clinical studies, animal studies, case reports, reviews, and studies with insufficient data, non-English publications, or irretrievable full texts. Only studies published in English were included to ensure accurate interpretation and synthesis of findings.

**Table 1:** PICOS framework used to determine eligibility of the articles in the present systematic review PICO: Patient/Population, Intervention, Comparison, and Outcome

Component	Inclusion Criteria	Exclusion Criteria
Population	Human-extracted teeth used in in-vitro settings	Animal teeth; in-vivo clinical studies; teeth with caries, fractures, restorations, hypoplasia, or fluorosis
Intervention	Restoration with Cention or Cention N	Other restorative materials not involving Cention
Comparison	Restoration with conventional glass ionomer cement (GIC)	Comparisons not involving conventional GIC (e.g., composite only, other alkasites) unless Cention vs GIC data were extractable
Outcome	Quantitative evaluation of microleakage (e.g., dye penetration, stereomicroscopy)	Outcomes unrelated to microleakage (e.g., compressive strength, wear resistance)
Study Design	In-vitro laboratory studies	Clinical trials (RCTs, cohort, case-control), case reports, reviews, conference abstracts, editorials, letters, and studies not available in English

During the search, no randomized controlled trials, cohort studies, or other in-vivo clinical evidence evaluating microleakage of Cention versus GIC were identified. Only in-vitro studies fulfilled the inclusion criteria. This reflects the current state of the literature, where clinical trials on this topic are lacking.

Study Selection

Following the execution of the search strategy across multiple databases and supplementary sources, all retrieved records were exported into reference management software for the removal of duplicates. The remaining articles were screened in two phases: an initial title and abstract screening, followed by full-text evaluation for eligibility.

Two independent reviewers screened the titles and abstracts of all identified studies to exclude irrelevant records. Studies were considered potentially eligible if they were in-vitro investigations conducted on human-extracted teeth comparing the microleakage of restorations using Cention versus conventional GIC. Full texts of these shortlisted studies were then retrieved and assessed independently by the same reviewers against the predefined inclusion and exclusion criteria framed using the PICOS model.

Agreement between reviewers during both title/abstract and full-text screening was high (>90%). Any discrepancies were discussed and resolved through consensus, with a third reviewer consulted when necessary. Cohen’s kappa coefficient (κ = 0.86) was calculated to quantify inter-reviewer reliability, indicating strong agreement. Ultimately, ten studies meeting all criteria were included in the final synthesis and meta-analysis. Potentially relevant clinical trials, animal studies, and case reports were excluded as per predefined criteria, and no ongoing registered clinical trials were identified during protocol development. Therefore, the review is exhaustive within the available in-vitro evidence base.

Outcome Measures

The primary outcome parameter assessed in this review was the degree of microleakage at the tooth-restoration interface following placement of Cention and conventional GIC restorations. This was evaluated using validated dye penetration techniques visualized under stereomicroscopy and graded according to established scoring systems.

Secondary outcome parameters included the influence of variables such as the type of cavity (Class I, II, or V), use of adhesives with Cention, and thermocycling protocols on the extent of microleakage. These factors were analyzed qualitatively to explore patterns of variation across studies.

Data Extraction

A standardized data extraction form was used to collect relevant information from the included studies. The extracted data included study characteristics (authors, publication year, study design), samples used for the study, types of interventions (Cention and conventional GIC), preparatory details (sectioning, thermocycling), microleakage assessment methods, outcomes, and conclusive findings.

Quality Assessment of the Included Studies

Since all the included studies were of in-vitro design, the QUIN tool was used for assessing the risk of bias [9]. The study’s quality assessment was conducted according to a set of domains of bias, and the final assessment was performed by categorizing each of the study features as “low”, “unclear”, or “high” risk of bias. The cumulative scores were subsequently employed to categorize the in-vitro study into one of three risk levels: high (<50%), medium (50-70%), or low risk (>70%). The categorization was performed as the total score × 100 divided by twice the number of criteria applicable.

Meta-analysis, Assessment of Heterogeneity, and Publication Bias

The standardized mean difference (SDM) with 95% CI was calculated for continuous outcomes. A fixed effects model (Mantel-Haenszel method) was used if there was no heterogeneity (p >0.05 or I-squared ≤24%); otherwise, a random effects model (Der Simonian- Laird method) was used [10]. All statistical analyses were performed using RevMan 5.3. The significance level was kept at p<0.05.

The significance of any discrepancies in the estimates of the treatment effects of the different trials was assessed by means of Cochran’s test for heterogeneity and the I2 statistics. Heterogeneity was considered statistically significant if P < 0.1. To test for the presence of publication bias, the relative symmetry of the individual study estimates was assessed around the overall estimates using Begg’s funnel plot.

Results

**Figure 1:**
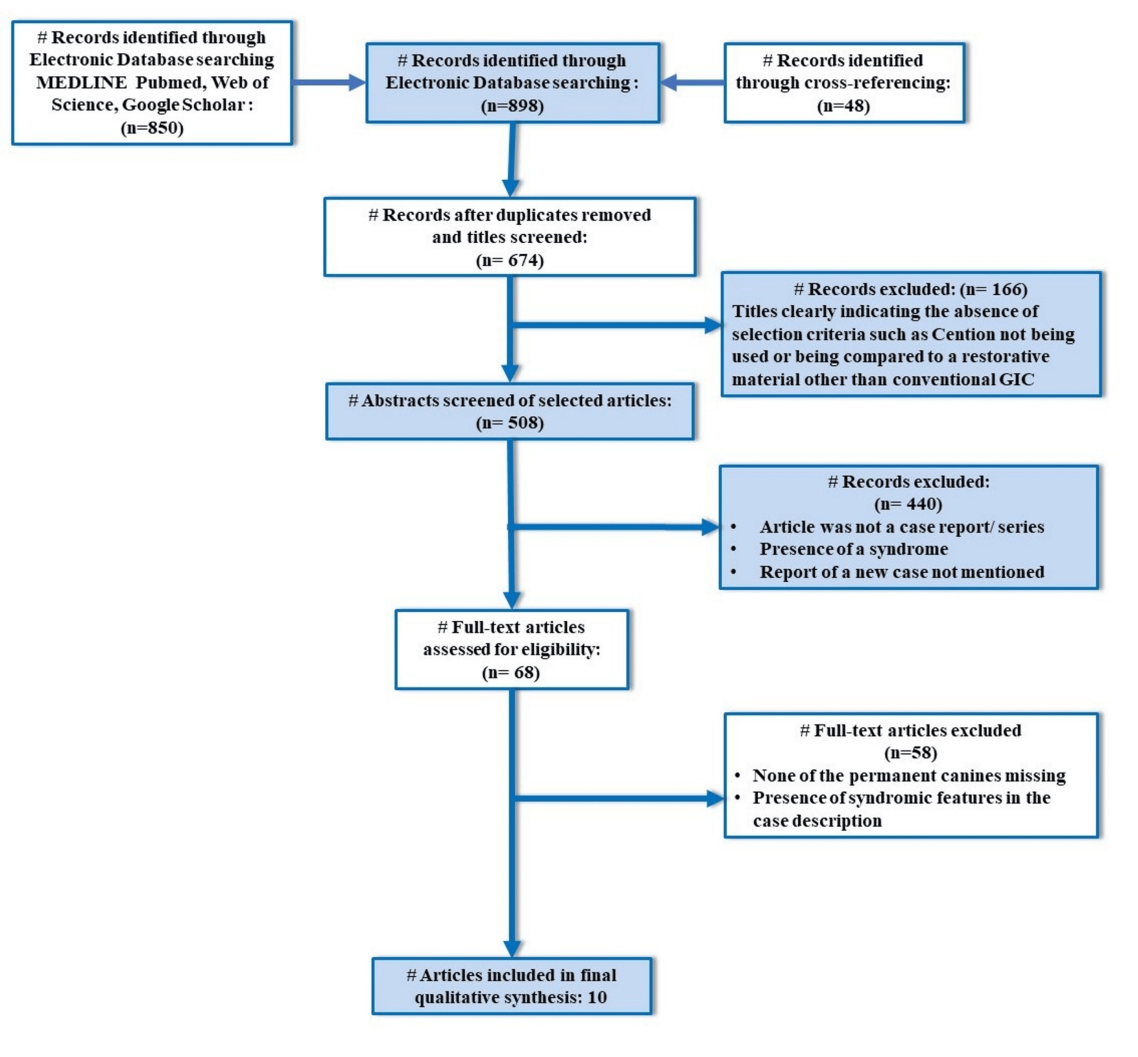
PRISMA flowchart indicating the selection process of the articles in the present systematic review # = number of records

In the present systematic review, n=10 studies (Figure 1) were identified, the earliest of which was published in the year 2018 and the latest study in 2021 [6,11-18]. The data extracted from these studies are comprehensively summarized in Table 2.

**Table 2:** Data related to study characteristics and outcomes of the included studies CN: Cention N; GIC: glass ionomer cement; MD: mesiodistal; BL: buccolingual; RMGIC: resin-modified glass ionomer cement; ×: times (magnification); °C: degrees Celsius; mm: millimeters; wt: weight; h: hour(s) Unpublished data: Ashok L. A comparative evaluation of microleakage around class V cavities restored with five different tooth coloured restorative materials – an in vitro study [dissertation]. Chennai: The Tamil Nadu Dr. M.G.R. Medical University; 2020

Sr. No.	Author	Year	Sample Size	Tooth	Inclusion Criteria	Exclusion Criteria	Preparation	Size	Comparison Between	Processing	Dye Used	Direction of Sectioning	Method of Observation	Scoring of Microleakage	Conclusive Findings
1	George and Bhandary [6]	2018	50	Premolars	Extracted for orthodontic purposes	Presence of caries	Class II cavity	2 × 2 × 1.5 mm	Group 1– Amalgam Group 2 - GIC Group 3 –Packable Composite Group 4 – Cention N Without Adhesive Group 5 – Cention N With Adhesive	Sored in distilled water at 37°c for 24 hours, following which, they were subjected to a thermocycling regimen of 500 cycles between 5°c and 60°c, with a dwell time of 20 seconds in each bath.	0.1% methylene blue for 24 hours	MD	Stereomicroscope	Degree 0: no dye penetration. Degree 1: up to 1/2 the gingival seat. Degree 2: >1/2 the gingival seat. Degree 3: all along the gingival seat. Degree 4: degree 3 plus into the axial wall.	• The mean microleakage among the various groups was seen to be highest with GIC restorations. • Cention N showed lesser microleakage compared to GIC and composite restorations. • Groups restored using Cention N without adhesive showed less microleakage compared to those with adhesive.
2	Kaur et al. [11]	2018	30	Premolars	Extracted non-carious premolars	Presence of caries	Class V Cavity	3 × 2 × 2 mm	Cention and Glass Ionomer Cement	Thermocycling between 5°C ± 4°C and 55°C ± 4°C for 500 temperature cycles	0.5% methylene blue for 24 hours	BL	Stereomicroscope	0: No leakage in combination with a stable, efficient self-cure initiator, 1 : Less than and up to one-half of the depth of the cavity preparation penetrated by the dye restoration7 2 : More than one-half of the depth of the cavity preparation penetrated by the dye but not up to the junction of the axial and occlusal or gingival wall 3 : Dye penetration up to the junction of the axial and occlusal or gingival wall but not including the axial wall 4 : Dye penetration including the axial wall	Conventional GIC exhibited the highest microleakage, and the least microleakage was shown by Cention N.
3	Kini et al. [12]	2019	24	Premolars	Human maxillary first permanent premolars with intact occlusal surfaces extracted for orthodontic purposes	Presence of caries and Fluorosis	Class I cavities	0.8 × 1.5 mm	Group I: Cention N without adhesive, group II: Cention with adhesive, group III: type IX glass ionomer cement, group IV: posterior composite	Thermocycling at temperature baths of 5°C, 37°C, and 55°C, with a dwell time of 30 seconds in each bath.	1% aqueous solution of the methylene blue dye for 24 hours	MD	Stereomicroscope	0° = no leakage 1° = less than or up to one-half of the depth of the cavity preparation 2° = more than one-half of the cavity preparation involved, but not up to the junction of the axial and occlusal or cervical wall 3° = dye penetration up to the junction of the axial and occlusal or cervical wall, but not including the axial wall 4° = dye penetration including the axial wall	• None of the materials was completely free of microleakage. • The cavities restored with Cention N, following application with an adhesive, showed the least microleakage among all groups.
4	Mazumdar et al. [13]	2019	30	mandibular first molars	Extracted recently	• Presence of caries • Restored teeth	Class II Cavity	Occlusal floor ‑ width 4 mm, length 5 mm; axial wall ‑ width 4 mm, height 3 mm; gingival floor ‑ width 4 mm, depth 2.5 mm	Group I‑silver Amalgam Group II‑Type II GIC Group III‑Cention - N	Thermocycled for 500 cycles between 5°C and 55°C with a dwell time of 30 s in each bath.	0.5% basic fuchsin dye for 24 h	MD	Stereomicroscope	no dye penetration 0.00 dye penetration up to the first‑third of the prepared cavity wall 0.25 dye penetration up to the second‑third of the prepared cavity wall 0.50 dye penetration into the entire prepared cavity wall 0.75 dye penetration into the entire prepared cavity wall and the pulpal wall 1.0	• All the restorative materials used in the study were unable to prevent the microleakage completely. • Out of all the restorative materials, CN, a newer restorative material, displayed minimum microleakage compared to AA and GICs.
5	Naz et al. [14]	2019	45	Premolars	Extracted for orthodontic purposes	Presence of caries	Class V cavity	3 × 2 × 1.5 mm	GIC type IX, Zirconomer Improved and Cention N	Performed, but details not specified	2% methylene blue dye for 48 hours	BL	Stereomicroscope	0 = No dye penetration. 1 = Dye penetration up to 1/3rd cavity depth 2 = Dye penetration up to 2/3rd cavity depth 3 = Dye penetration to full depth of cavity 4= Dye penetration onto axial wall of cavity.	• The mean microleakage score was minimum in Cention N, followed by Zirconomer Improved, and was maximum in GIC type IX.
6	Punathil et al. [15]	2019	60	Primary Molars	Extracted for various therapeutic reasons	Carious, fractured, or previously restored teeth	Class II mesioclusal cavity	2 × 2 × 1.5 mm	Nano-filled, resin-modified, glass-ionomer, nanocomposite resin, and Cention N	Thermocycling (100 cycles) 5°C, 30 seconds; 19°C, 20 seconds; 55°C,30 seconds	submerged for a period of 1 day at 37°C in the basic fuchsin dye solution (0.5%)	MD	Stereomicroscope (×40 magnification)	Score 0: No microleakage Score 1: Dye penetration up to one-third of the axial wall Score 2: Dye penetration up to two-thirds of the axial wall Score 3: Dye penetration onto the entire axial wall Score 4: Dye penetration onto the pulpal wall.	• The lowest microleakage was found to be associated with teeth restored by the nano-filled RMGIC group, followed by the Cention N group and the nanocomposite resin group.
7	Aakriti et al. [16]	2020	30	Premolars	extracted for orthodontic causes	Carious teeth	Class V cavity	3 × 2 × 2 mm	Conventional glass ionomer cement, Equia Forte, Cention N	Thermocycling between 5ºC ± 4ºC and 55ºC ± 4ºC for 500 temperature cycles.	0.5% Methylene blue dye for 24 h	BL	Stereomicroscope	0=No leakage, 1=Less than and up to one-half of the depth of the cavity preparation penetrated by the dye 2=More than one-half of the depth of the cavity preparation penetrated by the dye, but not up to the junction of the axial and occlusal or gingival wall 3=Dye penetration up to the junction of the axial and occlusal or gingival wall, but not including the axial wall 4=Dye penetration, including the axial wall	• None of the three restorative materials compared was free from microleakage. • Cention N displayed the least microleakage and came to be better than the Equia Forte and Conventional GIC
8	Ashok (unpublished literature)	2020	30	Maxillary premolars	extracted for orthodontic purposes	Teeth with caries, cracks, or restorations	Class V cavity	4 × 2 × 4 mm	Conventional GIC, Zirconomer, Giomer, Cention N, and Ionoseal	Thermocycling between 5°C and 55±2°C for 500 cycles with a dwelling time of 15 seconds.	50% Wt silver nitrate dye for 6 hours at room temperature	BL	Stereomicroscope (×30 magnification)	0 = No microleakage, No dye penetration 1 = Microleakage observed only at the cavity wall of enamel, Dye penetration through the cavity margin reaching the enamel or cementum 2 = Microleakage observed at the cavity wall of dentin but not on the cavity floor, Dye penetration through the cavity margin reaching the dentin 3 = Microleakage observed on the cavity floor, Dye penetration through the cavity margin reaching the cavity floor	The order of microleakage from minimum to maximum, Ionoseal < Cention N < Giomer < Zirconomer < Conventional GIC respectively.
9	Sujith et al. [17]	2020	45	Premolars	Orthodontically extracted	Presence of a crack, caries, restoration, or white spot lesion on the buccal surface	Class V cavity	2 × 3 × 2 mm	Cention N, GIC, and hybrid composite	200 thermocycles at 5°C and 55°C lasted for 30 seconds	0.5% basic fuchsine dye at 37°C for 24 h	BL	Stereomicroscope (×20 magnification)	Score 0: No evidence of microleakage; Score 1: Penetration of dye up to half of cavity depth; Score 2: Microleakage more than half of the depth of cavity wall; Score 3: Leakage of dye involving axial wall.	Cention N exhibits lesser microleakage compared with GIC and composite.
10	Patil and Winnier [18]	2021	45	maxillary and mandibular first and second deciduous molars	Part of the serial extraction procedure, retained primary teeth were extracted at the time of eruption of permanent teeth	Grossly carious teeth or teeth with hypoplastic defects	Class II cavity	Not mentioned	Glass ionomer cement, Zirconomer, and Cention N	500 cycles of thermocycling between 5°C and 55°C with a dwell time of 30 seconds in each bath	2% methylene blue dye solution for 24 hours at 37°C	MD	Stereomicroscope (×22.5 magnification)	Score 0 = No dye penetration. Score 1 = Dye penetration into the enamel. Score 2 = Dye penetration into the dentin, not including the pulpal wall. Score 3 = Dye penetration into the dentin, including the pulpal wall.	• None of the restorative materials were able to completely prevent microleakage. • Among the three restorative materials, Cention N exhibited the least microleakage, followed by GIC and Zirconomer.

While the minimum criteria were that the studies should be comparing microleakage with Cention restorations to conventional GIC, some studies explored microleakage in other types of reinforced GIC and composite resins. Two studies also investigated the microleakage in Cention restorations with and without the use of adhesive systems. Seven out of ten studies used extracted premolars, two studies used deciduous molars, and one study used permanent molars. All the studies that used premolars included only those extracted for orthodontic purposes. The studies utilizing molars used teeth extracted as a part of any therapeutic procedure, such as during serial extraction or those shed during the eruption of permanent teeth.

Among the studies utilizing premolars, five studies prepared Class V cavities on the study teeth, and one prepared Class I cavities. All three studies utilized molars prepared with Class II cavities. The majority of the studies commonly adopted a cavity size of dimensions 3 mm × 2 mm × 2 mm or 2 mm × 2 mm × 1.5 mm [6,11-18]. To simulate the oral conditions, all the studies performed thermocycling between the temperature range of 5 to 55 °C, with a dwelling time of 15 to 30 seconds, and the number of cycles ranging from 100 to 500. Based on the conclusive findings presented by the authors of the studies included in the current systematic review, it is apparent that Cention, in general, exhibited lower microleakage when compared to GIC.

Assessment of Methodological Quality of Included Studies

**Figure 2:**
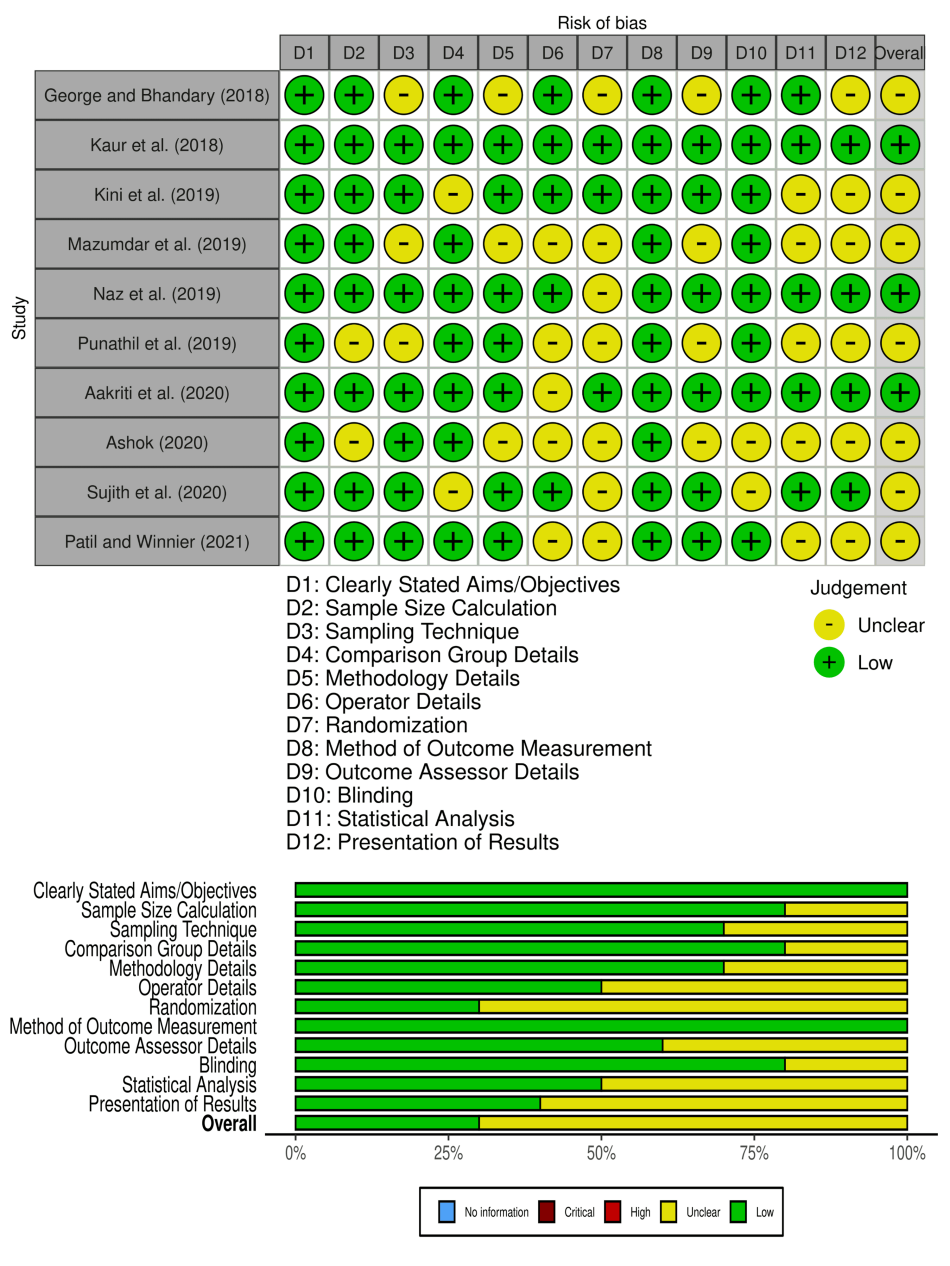
Risk of bias among the included studies References [6,11-18]

The methodological quality of the included studies was assessed using the QUIN tool. Most studies demonstrated a low to moderate risk of bias across the evaluated domains (Figure 2). A higher risk of bias was noted in domains such as randomization, blinding of outcome assessors, and statistical analysis, reflecting areas that require methodological improvement. Conversely, domains like clearly stated objectives, detailed methodology, and outcome measurement exhibited a consistently low risk of bias across studies.

Among the included studies, Mazumdar et al. (2019) exhibited the highest overall risk of bias, with medium risk scores across multiple domains, such as sampling technique and operator details. On the other hand, studies like Kaur et al. (2018), Naz et al. (2019), and Aakriti et al. (2020) demonstrated low overall risk, reflecting robust methodological standards in most domains [11,13,15,16].

Domains like sample size calculation, operator details, and randomization were among the more frequently reported sources of moderate bias, whereas criteria such as blinding of participants, presentation of results, and statistical analysis often displayed low levels of bias. The overall quality assessment highlights the need for enhanced adherence to rigorous methodological standards in future studies.

Meta-analysis

The SMD was used as a summary statistic measure for continuous outcomes. The outcomes were assessed in terms of better efficacy between conventional GIC and Cention in terms of its effect on microleakage. Data was evaluated from eight studies from an aggregate of 210 samples, of which 105 samples were evaluated by conventional GIC and 105 samples were evaluated by Cention for the evaluation of the better efficacy between the two restorative materials in terms of lesser microleakage as an outcome.

**Figure 3:**
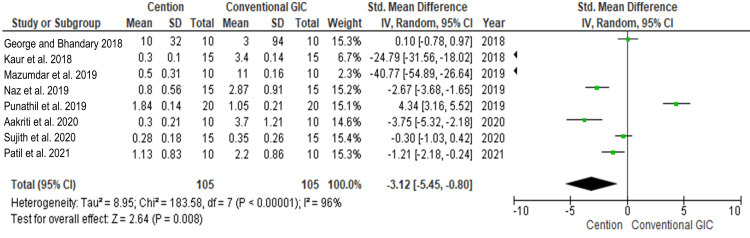
Forest plot showing the comparison between Cention and conventional GIC with regard to lesser microleakage. References [6,11-18]

As shown in Figure 3, the SMD is -3.12 (-5.45 - 0.80) and the pooled estimates favor the Cention group. This signifies that the reduction in microleakage on average was 3.12 times more in the Cention group compared to the conventional GIC, and the difference was not statistically significant (p>0.05). Among the included studies, the highest weightage was given by Sujith et al. 2020 while the lowest weightage of the study was given by Mazumdar et al. 2019 [13,17]. By employing the random effect model, the I2 statistic showed 96%, the heterogeneity for Tau2 was 8.95, x2 being p<0.005, and the overall effect for Z value was 2.64 (P=0.008).

**Figure 4:**
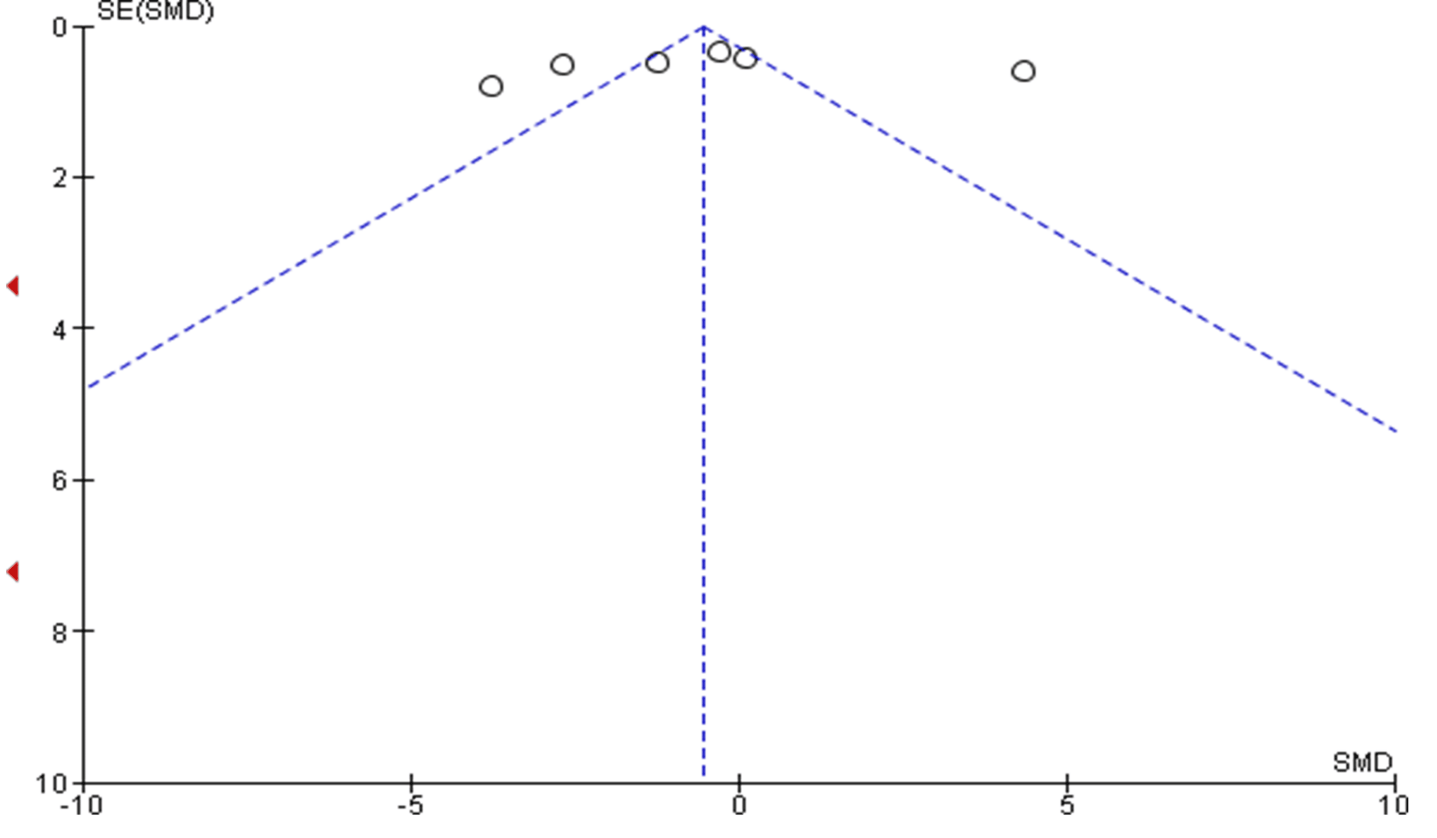
Begg’s Funnel plot with 95% confidence intervals demonstrating asymmetric distribution without systematic heterogeneity of individual study compared with the standard error of each study, indicating the presence of publication bias.

The funnel plot did show significant asymmetry, indicating the presence of publication bias (Figure 4). The funnel plot showing asymmetric distribution with the presence of systematic heterogeneity of individual studies compared to the standard error indicated the presence of publication bias in the meta-analysis.

Discussion

Tooth and Cavity Selection

The primary objective of this systematic review was to assess and synthesize the existing literature comparing microleakage after restoration with Cention and conventional GIC in human extracted teeth. Ten studies published between 2018 and 2021 were included, reflecting the relatively recent introduction of Cention into dental practice [6,11-18].

Most studies used extracted premolars (n=7), while two employed deciduous molars and one used permanent molars. Premolars are frequently chosen due to their common extraction during orthodontic treatment [19], whereas molars, particularly deciduous ones, are more often affected by caries and are easier to procure [20,21]. The use of multiple tooth types enhances the generalizability of results across different clinical contexts. Importantly, all studies excluded teeth with caries, fractures, restorations, hypoplasia, or fluorosis to ensure standardized, structurally sound samples [22-25]. This methodological choice minimized confounding and allowed the focus to remain on restorative material performance.

Cavity preparation also varied: most studies evaluated Class V restorations, while Class I and Class II cavities were also included. Class V cavities are clinically relevant for cervical restorations with high microleakage risk [26], Class I cavities offer baseline comparability [27], and Class II cavities represent common clinical scenarios on proximal surfaces [28]. This diversity provides a broader context for evaluating restorative performance.

The majority of the studies commonly adopted a cavity size of dimensions 3 mm × 2 mm × 2 mm or 2 mm × 2 mm × 1.5 mm. These cavity sizes are often chosen because they approximate the dimensions of cavities encountered in real clinical practice. Such a cavity size corresponds to a moderate-sized restoration that a dentist may commonly encounter when treating carious lesions [29]. Standardized dimensions ensure that the cavity preparation process is uniform, reducing variability and allowing for a more reliable comparison between Cention restoration and conventional GIC. This made the analysis of the findings more homogeneous in the present systematic review by reducing confounding due to dimensions. It can thus be interpreted with confidence that the discrepancies in material performances were not due to variations in cavity size.

Thermocycling Protocols

To simulate intraoral conditions, all studies incorporated thermocycling. Despite some variation, protocols were consistent: temperature ranges between 5°C and 55°C, dwell times of 15-30 seconds, and cycle counts from 100 to 500 [30-32]. These parameters replicate the thermal stresses of consuming hot and cold foods and beverages and approximate clinical conditions. Standardization of these protocols strengthened the reliability of cross-study comparisons.

Microleakage Assessment Methods

Microleakage was uniformly assessed using stereomicroscopy, providing high-resolution visualization of the tooth-restoration interface [32]. Preparatory steps, such as sectioning and thinning, optimized visualization [33]. Most studies employed graded scales based on the thirds of cavity preparation, although one evaluated leakage by enamel, dentin, and pulp involvement [16]. While this discrepancy reduced scale homogeneity, overall comparability was maintained. Consistency in methodology enhances the validity of pooled results [34,35].

Comparative Performance of Cention vs GIC

Meta-analysis demonstrated that Cention exhibited significantly lower microleakage than conventional GIC, with leakage reduction 3.12 times more prominent in the Cention group. The study by Sujith et al. (2020) contributed the highest weightage, while Majumdar et al. (2019) had the lowest [13,17]. Funnel plot analysis revealed asymmetry, suggesting publication bias; studies with positive findings may be preferentially published.

Exceptions were noted. One study found Ionoseal superior to Cention [12], and another reported nano-filled RMGIC outperforming Cention [17]. These results suggest that while Cention generally performs better, alternative materials may be preferable in specific clinical contexts.

Limitations of the Evidence

Several limitations must be acknowledged. First, all included studies were in-vitro, limiting extrapolation to clinical practice where saliva, occlusal forces, pH fluctuations, and biofilm interactions can influence microleakage. Second, heterogeneity existed in cavity classes, tooth types, cavity dimensions, use of adhesives, and thermocycling protocols, reducing direct comparability. Third, although efforts were made to harmonize outcomes, minor differences in scoring systems introduced variability. Fourth, funnel plot asymmetry indicated significant publication bias, raising concerns that negative or neutral studies may be underrepresented. Finally, the small number of eligible studies and modest sample sizes reduce statistical power and limit the strength of conclusions.

Importantly, no in-vivo or clinical studies were identified, and no ongoing trials appear to be registered. This highlights a major evidence gap: whether the in-vitro advantages of Cention translate into meaningful clinical outcomes remains unverified. Future randomized controlled trials with standardized protocols are urgently needed to validate laboratory findings and guide restorative material selection in practice.

## Conclusions

In the present systematic review, a total of 10 studies spanning from 2018 to 2021 were reviewed to analyze microleakage in dental restorations involving Cention and other materials. The majority of the included studies in the present systematic review found that Cention tended to have lower microleakage compared to conventional GIC, supporting its effectiveness in reducing leakage at the tooth-restoration interface. However, the exceptions, where Ionoseal and nano-filled RMGIC were reported as superior in specific studies, suggest that the choice of restorative material should be influenced by the clinical context and the specific requirements of each case. The findings of the present systematic review contribute to our understanding of the potential of Cention as a reliable option in restorative dentistry, emphasizing the importance of evidence-based decision-making in dental practice, considering the properties and performance of different materials in various clinical scenarios.

The meta-analysis indicates a trend toward reduced microleakage in the Cention group. However, the lack of statistical significance and the presence of substantial heterogeneity and publication bias highlight the need for cautious interpretation. Future studies with larger sample sizes, standardized methodologies, and thorough reporting are essential to further elucidate the comparative performance of Cention and conventional GIC in dental restorations.

## Appendices

Search

((cention) AND (glass ionomer cement)) AND (microleakage)

"cention"[All Fields] AND ("glass ionomer cements"[MeSH Terms] OR ("glass"[All Fields] AND "ionomer"[All Fields] AND "cements"[All Fields]) OR "glass ionomer cements"[All Fields] OR ("glass"[All Fields] AND "ionomer"[All Fields] AND "cement"[All Fields]) OR "glass ionomer cement"[All Fields]) AND ("microleakage"[All Fields] OR "microleakages"[All Fields])

Translations

Glass Ionomer Cement

"glass ionomer cements"[MeSH Terms] OR ("glass"[All Fields] AND "ionomer"[All Fields] AND "cements"[All Fields]) OR "glass ionomer cements"[All Fields] OR ("glass"[All Fields] AND "ionomer"[All Fields] AND "cement"[All Fields]) OR "glass ionomer cement"[All Fields]

Microleakage

"microleakage"[All Fields] OR "microleakages"[All Fields]
